# New Evaluation of Postovulatory Follicle Degeneration at High-Temperature Regimes Refines Criteria for the Identification of Spawning Cohorts in the European Anchovy (*Engraulis encrasicolus*)

**DOI:** 10.3390/ani11020529

**Published:** 2021-02-18

**Authors:** Rosalia Ferreri, Marco Barra, Antonella Gargano, Salvatore Aronica, Angelo Bonanno, Simona Genovese, Paola Rumolo, Gualtiero Basilone

**Affiliations:** 1 Consiglio Nazionale Delle Ricerche (CNR)—Istituto per lo Studio Degli Impatti Antropici e Sostenibilità in Ambiente Marino (IAS), SS Capo Granitola, Via del Mare, 3, 91021 Campobello di Mazara, Italy; antonella.gargano@ias.cnr.it (A.G.); salvatore.aronica@cnr.it (S.A.); angelo.bonanno@cnr.it (A.B.); simona.genovese@cnr.it (S.G.); gualtiero.basilone@cnr.it (G.B.); 2 Istituto di Scienze Marine—Consiglio Nazionale delle Ricerche, Calata Porta Di Massa—Porto Di Napoli 80, 80133 Napoli, Italy; marco.barra@cnr.it (M.B.); paola.rumolo@cnr.it (P.R.)

**Keywords:** histology, postovulatory follicle size, resorption rate, daily spawning cohort, European anchovy

## Abstract

**Simple Summary:**

Wide fluctuations in abundance arising from variations in reproductive success are characteristic of many marine fish populations, including several pelagic fish species. The European anchovy is an economically and ecologically important resource, representing one of the most abundant species of the total Mediterranean fishery production. According to recommendations by the European Union, management plans should improve estimates of model parameters or biomass evaluation to provide a sustainable stock exploitation. The proportion of females spawning per day is one of the main parameters, including ichthyoplankton methods, for spawning biomass evaluation and is usually assessed by the postovulatory follicle (POF) method. Because of POF resorption rate is species-specific and influenced by residence temperature, the application of this method needs validation for species and area. This manuscript aimed to evaluate the resorption rate of postovulatory follicles in European anchovy in the central Mediterranean Sea, describing each degeneration stage based upon its histological features and estimating the duration of each POF stage in relation to water temperature experienced by the target species during the spawning peak. The provided results should allow for methodological advances in estimating the spawning biomass and in studying of reproductive output fluctuations, particularly for sustainable exploitation purposes.

**Abstract:**

Accurate stock assessment estimates of fish resources are essential in fishery management. Wide fluctuations in abundance arising from variations in reproductive success are characteristic of many marine fish populations, including multiple spawner species. The proportion of females spawning per day is crucial in the application of egg production methods for spawning biomass evaluation and, usually, is assessed by postovulatory follicle (POF) method. Describing each degeneration stage of POF based upon its histological features allows for obtaining an aging key for postovulatory follicles. The commercially valuable European anchovy (*Engraulis encrasicolus*) was selected as a case study, which breeds during the summer in temperate waters (24 °C–25 °C). A collection of ovary slides, sampled in the central Mediterranean Sea during the spawning peak, provided a 24 h cycle coverage. These observations allowed us to evaluate the duration of each POF stage at water temperature experienced by anchovy in the study area. Present results demonstrated the POF degeneration progress at a faster rate than reported by previous investigations, carried out in cooler oceanic waters. Furthermore, the present study displayed the presence of two anchovy spawning cohorts sampled along a 24-h cycle. Therefore, this study not only provides useful insight for more accurate POF degeneration evaluation in temperate waters, but also suggests that current estimates should be complemented with validation studies according to different temperature regimes.

## 1. Introduction

Wide fluctuations in abundance arising from variations in reproductive success are characteristic of many marine fish populations, including several small pelagic fish species (i.e., [1,2,3]). In modern natural resource management and particularly in the case of fisheries, evaluation of reproductive potential as well as spawning stock biomass are among the most important practical components of sustainability plans and ecosystem services management [4]. The ichthyoplanktonic methods allow to estimate the size of the spawning population, when are known how many eggs have been spawned over a period of time (e.g., daily or annually) in the spawning area and how many eggs an average individual mature female can produce over the same period [5]. The proportion of females spawning per day (i.e., the spawning fraction) is one of the main parameters for these estimates and, usually, it is assessed by postovulatory follicle (POF) method [6,7]. 

Hunter and Goldberg [6] introduced the POF method at the beginning of the 80s to estimate the spawning fraction in wild populations of the Northern anchovy (*Engraulis mordax*), but now it is applied to other multiple spawning clupeids—e.g., Peruvian anchovy (*Engraulis ringens*), European anchovy (*Engraulis encrasicolus*), sardine (*Sardina pilschardus*) [8,9]—and to a large number of other fish taxa—e.g., sprat (*Sprattus sprattus*), mackerel (*Scomber scombrus*) and snapper (*Pagrus auratus*) [9]. Despite its popularity, the POF method application can be affected by some sources of bias, such as POF staging and ageing or wrong sampling design [7]. Because the POF resorption rate is species-specific and influenced by residence temperature, the application of this method needs a validation for species and area, i.e., an accurate aging key, identifying the stages by definite histological features and describing the temporal trend on POF degeneration [7,10,11,12,13].

Prior to ovulation, the follicle comprises two layers of cells surrounding the developing egg, the outer thecal and inner granulosa, with prominent nuclei and regular columnar shape [14]. POFs are produced in cohorts following each ovulation event, according to the inter-spawning interval [15]. The morphology of POFs should be studied in detail in relation to the time of collection, daily spawning period, and existing descriptions of POFs for other anchovy stocks or species. Indeed, several studies confirmed the POF ageing was possible, linking POF histomorphological structure to the time after the spawning, by the examination of a series of field-collected ovaries [7,10,16]. When involving field samples, a prerequisite for this study is that the target population exhibits daily spawning synchronicity, which is the case of European anchovy [9,17,18].

The European anchovy is an economically and ecologically important resource, representing together with other small pelagic species like the sardine, the most abundant species of the total Mediterranean fishery production [19]. According to recommendations by the Scientific, Technical and Economic Committee for Fisheries of the European Union (STECF) [20] and the General Fisheries Commission for the Mediterranean (GFCM), management plans should improve estimates of model parameters or biomass evaluation to provide sustainable stock exploitation [21,22]. In the Strait of Sicily, a long-time series of acoustic and ichthyoplankton-based biomass evaluation showed high inter-annual fluctuations in biomass, especially linked to environmental variability [3,23,24].

Previous studies, focused on anchovy POF duration vs. temperature, were performed within different temperature ranges [11,25,26]. Recent studies showed criteria for the classification of POFs and their aging, specifically developed for anchovy in the Atlantic waters [13,27]. Despite some attempts to adopt such criteria for the Mediterranean [28], no studies have been carried out to date on field individuals in the central Mediterranean Sea during the summer season, when anchovy spawning reaches its peak [29]. 

The aim of this paper is to evaluate the resorption rate of postovulatory follicles, by a 24 h covering collection of ovary slides obtained by field adults of European anchovy, sampled in the Strait of Sicily during the spawning peak. Firstly, describing each degeneration stage based upon their histological features; later, these observations were employed to evaluate the duration of each POF stage in relation to the daily spawning peak and time of capture, as well as the variation of the resorption rate among different POF stages.

## 2. Materials and Methods

### 2.1. Field Sampling

No use of live animals has been required for this study and no specific permissions were needed for the sampling activities in the investigated area because the target species is commercially harvested (neither endangered nor protected) and it was caught in areas where fishing is allowed.

**Figure 1:**
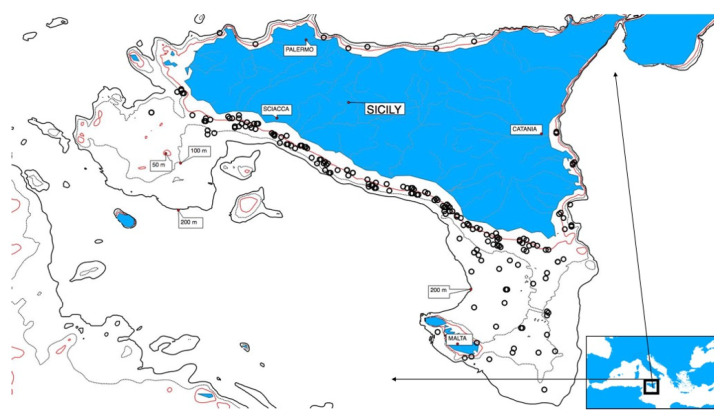
Locations of European anchovy sampling performed on the continental shelf in the Strait of Sicily.

Combined daily egg production method (DEPM) [30] and echo-acoustic surveys were accomplished on board a research vessel equipped with a midwater pelagic trawl, with the aim to evaluate abundance and distribution of small pelagic fish species [3,23,24,31]. In 2008 and 2009, additional opportunistic samples were obtained on board of commercial fishing vessels during the same period of DEPM surveys [32]. Adult individuals of European anchovy were caught in the Strait of Sicily (Figure 1), within the main spawning sites in the study area [33]. The sampling was carried out in 2008, 2009, and from 2012 to 2015 during the anchovy spawning peak (June–August [29]) (Table 1). During the sampling period, the average sea surface temperature in the study area was in the range between 23.5 °C and 25.5 °C [24]. On average, samples consisted of a random collection of 738 ± 361 g of anchovy, each containing a maximum of 75 individuals. On board, fish were sexed immediately after capture, and ovaries were preserved in 4% buffered formalin for further histological analysis. 

### 2.2. Laboratory Analysis

**Figure 2:**
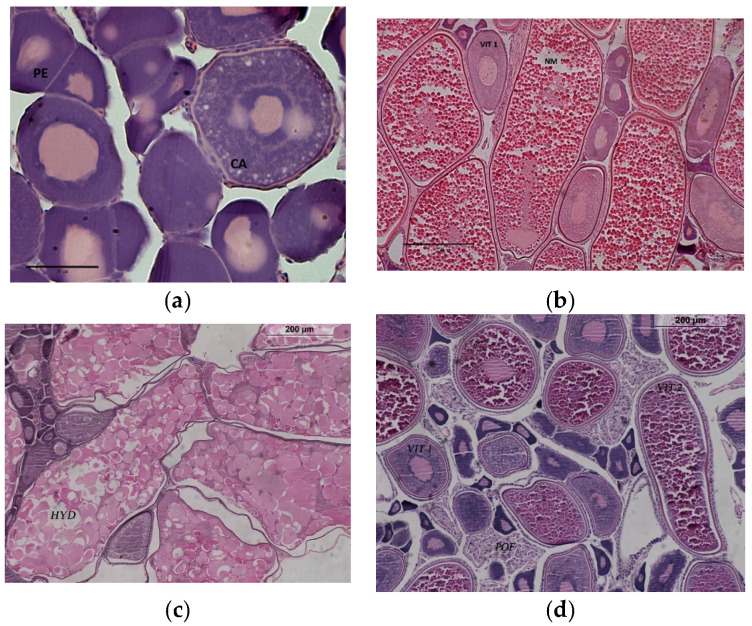
Histological sections of anchovy ovary. Different reproductive phases were displayed: (**a**) Developing; (**b**) Imminent spawning; (**c**) Spawning; (**d**) Partial post-spawning (defined according to Ferreri et al. [34]). Different oocyte development stages were also highlighted. PE = perinucleolar oocyte stage; CA = cortical alveoli; VIT1 = partially vitellogenic oocytes; VIT 2 = fully vitellogenic oocytes; NM = nucleus migration; HYD = hydrated oocytes; POF = post-ovulatory follicle. Scale bar: image “a” bar = 50 μm; images “b–d” bar = 200 μm.

A small part of ovarian tissue was cut from each field-collected ovary. Tissue subsamples were dehydrated and cleared in xylol by an automated sample tissue processing system (Leica, TP1020), then embedded in paraffin and four-micron histological sections were obtained, using an automatic rotary microtome (Leica RM2255). Finally, the ovary slides were stained with haematoxylin-eosin (H&E). The scoring of histological preparations included the developmental stage of the advanced group of oocytes for the spawning phase definition, as described in Ferreri et al. [34] (Figure 2), and the presence and morphological characteristics of postovulatory follicles (POFs). A dataset composed of 245 ovary slides showing POFs was used for further analyses (Table 1). When POFs were identified, slides were scanned in detail for scrutinising the follicles at higher magnification (40×) to perform staging. Each stage was proposed referring to a certain degree of POF degeneration, characterized by morphologically identifiable features, among these commonly described in the literature (e.g., [11,27]).

The whole ovary histological sections were photographed using a digital camera (Leica, DFC 425) linked to a stereo microscope (Leica, MZ6). The pictures of each POF were carried out at 40× magnification using a digital camera (Leica, DFC 425) linked to an optical microscope (Leica, DM 2500). After being split per stage, measures of area were performed for each identified POF. The mean area (μm^2^) per POF stage and relative occupied area by POFs on the sectioned ovary tissue (mm^2^POF/mm^2^gonad tissue) were measured on stored images by an image analysis software (Leica Application Suite). The difference in mean size per stage was tested by the Kruskal-Wallis test (*p* ≤ 0.05 was considered significant), while the relative area was normalized per two hour-long daytime intervals. Residence temperature is acknowledged to affect the resorption rate; thus, the literature suggests different sampling schemes according to environmental characteristics. At moderate temperatures (around 16 °C) individuals should be sampled at least at 4 h lag, while at higher temperatures (28 °C–30 °C) sampling should be carried out at 1–2 h intervals [11]. Taking into account the anchovy reproductive strategy as well as the mean surface temperature experienced by anchovy in the study area (24 °C–25 °C; [24]), 2 h were considered the most appropriate interval for investigating the POF degeneration in field individuals [11].

### 2.3. Age Estimate

In order to reduce potential bias due to differences among the number of fishes sampled along the 24 h cycle (Table 2), the occurrence of each stage was standardized firstly by the total number of females with POFs and, then, by the total amount of POFs during the considered two hour-long daytime intervals. 

The time of capture and the daily spawning time were taken into account to estimate the age of POFs. Particularly, the mean age of each POF stage was estimated as the time in hours elapsed between the daily anchovy spawning peak (ASP; around 22.00; [17]) and the sampling time, using the standardized occurrence of POFs per two hour-long intervals. When a POF stage was detectable only in a portion of 24 h cycle, it was considered to belong to the youngest daily spawning cohort (Day 0). A POF stage present along the whole daily cycle, also showing peaks of minimum and maximum of presence, was considered to belong to a previous spawning cohort (Day 1). Therefore, when older stages are recorded at their minimum occurrence, also showing an overlapping with the youngest cohort, they were considered belonging to the oldest spawning cohort (i.e., two different cohorts) and the relative ages were obtained, adding 24 h (i.e., one day). 

## 3. Results

### 3.1. POF Stages Identification

**Figure 3:**
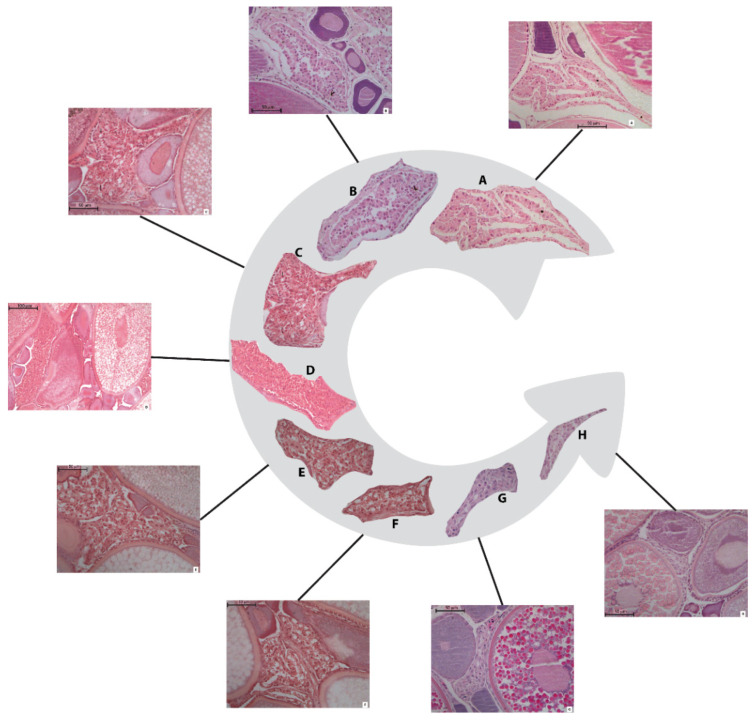
Evolution of postovulatory follicle (POF) resorption process (in the grey arrow). The rectangular panel showed each POF stage within the histological section of ovary. (**A**) POF I with no sign of degeneration; (**B**) POF I (BIS) with early evidence of degeneration; (**C**) POF II characterized by the appearance of vacuoles; (**D**) POF III showing size reduction and lumen shrinkage; (**E**) POF IV with compacted, flat aspect, folder and lumen almost not still visible (two POFs are distinguishable in the picture); (**F**) POF V shrunk appearance, lumen not visible, almost triangular-shaped (two POFs are distinguishable in the picture); (**G**) POF VI very small size, elongated shape; (**H**) POF VI (BIS) very late degeneration phase, with a triangular shape, representing the last appearance of recognisable POF. See Figure S1 for more details.

According to the histo-morphological criteria, six stages of POF resorption have been classified for the anchovy in the study area (Figure 3 and Figure S1).

Stage I: This stage showed no (Figure 3A) or very early (Figure 3B) evidence of degeneration. It is characterized by the presence of two cell layers, theca and granulosa, with still clearly visible nuclei and well-organized cells that determine flexible folders or a cordon-shaped structure. The granulosa cells are generally columnar or cubic. These cells displayed a good level of alignment, although they may not be rigorously compacted; moreover, they can still appear extended due to the extensive elongation experienced during the hydration phenomenon. The whole POF structure is strictly a folder, showing large size, irregular shape and irregular-shaped lumen (Figure 3A). This first stage of POF was detected in ovaries where oocytes showed partial or full vitellogenesis as the most advanced stage of development (Figure 2).

Stage II: The follicular structures appeared more wrinkled, although the mean size of POF II is very similar to the previous stage. This resorption phase is easily discriminated by the appearance of numerous vacuoles, affecting a large amount of the granulosa cells. Moreover, these cells changed their morphology, turning from columnar to a spread, spherical shape (Figure 3C). 

Stage III: The POFs belonging to this stage displayed a clear degenerated appearance, together with a reduction of extension and a lumen shrinkage until they are no longer recognizable. The structure acquired a flatter shape with only few residual folders. The theca cells form a thin layer, adhering to the granulosa. The numerous vacuoles enlarged, determining the breakdown in many of the cell walls and the onset of pycnotic phenomena in the cell nuclei (Figure 3D).

Stage IV: The structures assumed a compacted, flat aspect, and showed reduced extension, without folder and lumen in almost all of the POFs at this resorption stage. The theca layer is still visible, although its cells are not easily distinguishable. The vacuoles, still abundant and well visible, induce the complete breakdown of the cellular walls; all the nuclei appear pycnotic (Figure 3E). 

Stage V: These POFs had a very compact, shrunken appearance with lumen not visible and almost triangular-shaped. The vacuoles were almost completely missing, as well as the cell walls in the granulosa layer, but the nuclei are identifiable, although pycnotic. The theca may be occasionally present but not easily distinguishable and, generally, may result incorporated with the connective tissue stroma (Figure 3F). The ovaries showing this follicles resorption stage may contain oocytes with nuclear migration, as the most advance stage of development (Figure 2).

Stage VI: The POFs showed very small extension and, generally, an almost triangular, elongated shape (Figure 3G,H). The follicles became amorphous, disorganized, without clear, distinguishable cellular structures. Some small, empty spaces among the cells may be observable, as well as some pycnotic nucleus. No cellular differentiation is visible in the theca layer. As in the previous stages, the ovaries containing POFs VI may display oocytes with nuclear migration (Figure 2).

### 3.2. POF Size and Age

**Figure 4:**
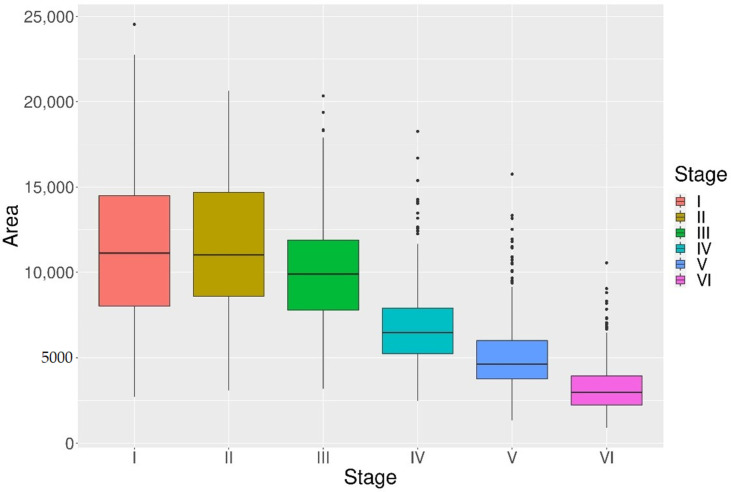
Mean area (μm^2^) of postovulatory follicles (POF), measured on stored images by an image analysis technique per each POF stage (I to VI).

The mean areas obtained by the image analysis of gonadic tissues showed the progressive degradation of POFs is associated with a proportional size reduction among stages (Figure 4). However, at the very beginning, the area reduction is not remarkable, i.e., POF II size is about 99% of the previous stage. The original mean size (POF I) is reduced more than by half at stage V and POFs VI appeared about 28% of the POFs I area. Although the decreasing trend is detectable also in the early stages (POFs I to POFs III), only POFs IV, V and VI resulted significantly smaller than the previous stages and different among them (H (5, n = 1940) = 1056.59, *p* < 0.001) (Figure 4). The differences in mean size were evaluated for each POF stages separately and results are shown in Table 3.

**Figure 5:**
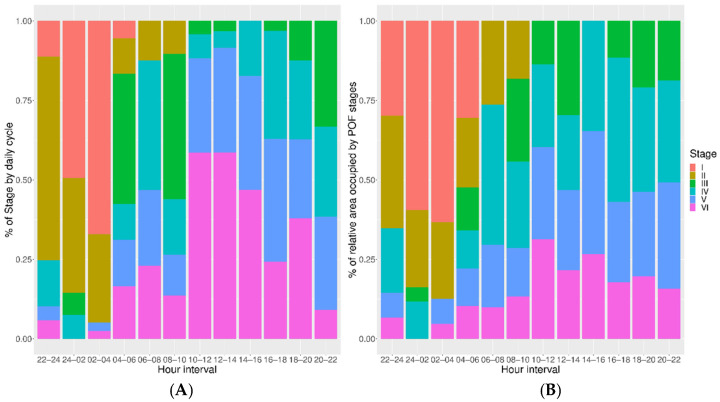
(**A**) Occurrence of postovulatory follicle (POF) stages (I to VI) standardized for the number of sampled females showing POFs in the ovaries, and (**B**) standardized average relative area occupied by POF stages (I to VI), per two hour-long intervals.

**Figure 6:**
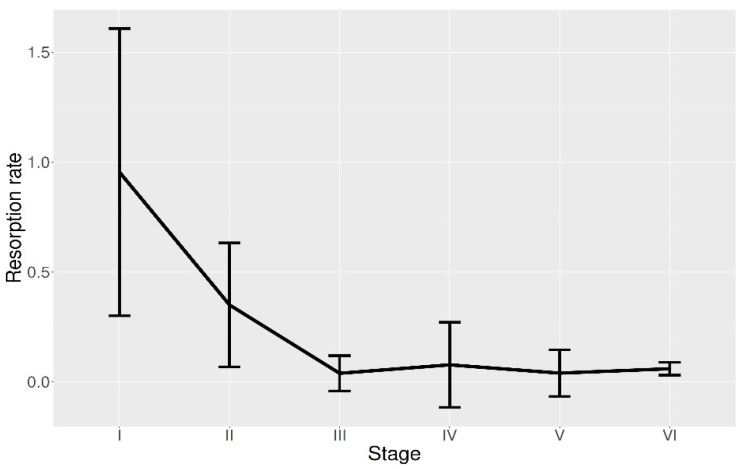
Mean resorption rate and relative confidence interval per each postovulatory follicle (POF) stage (I to VI), measured as area reduction along the two hour-long intervals.

The proportion in number and the relative occupied area per time intervals during 24 h were displayed for each POF resorption stage in Figure 5A,B, respectively. These patterns showed similar distributions of each POF stage according to a number of follicles or relative area. POF I displayed maximum abundance in both number and the relative area between 0:00–4:00. Figure 6 showed the trend of the resorption rate for each stage, by means of the evaluation of the total area occupied by each phase in relation to the ovary surface. This trend highlighted a more rapid contraction for stages I to III, although the reduction was progressive, with a more stable resorption rate from POFs III onwards. 

**Figure 7:**
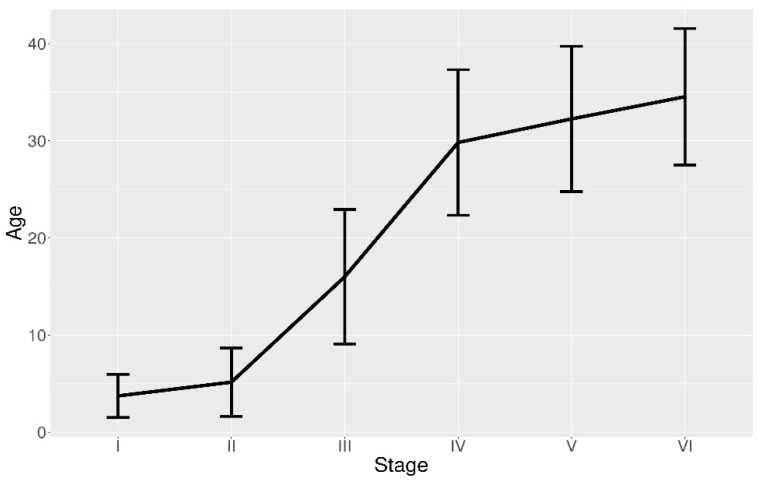
Average age (i.e., number of hours) and relative confidence interval of each postovulatory follicle (POF) stage (I to VI), measured by time between spawning time peak and time of capture.

The hourly distributions indicated that the early stages (POFs I to POF II) disappeared in about 10 h after the ASP. Meanwhile, POFs from stage III onwards were spread along the whole day, although displaying different incidence per two hour-long intervals and clear peaks of maximum and minimum abundance. However, all the resorption stages from IV to VI assumed lower values during night and morning. For POFs IV a peak of high presence was recorded in the interval from 6:00 to 8:00, while for stages V and VI similar peaks were observed later in the evening, starting in the early afternoon (Figure 5A,B). These distributions, which displayed only POFs I to III to persist less than one day (Figure 5A,B), look similar to POF stage aging (Figure 7). 

Stage I only occurred at certain times of the night close to the ASP, around 22:00 (local time) in the study area [17]. The follicle in this first resorption stage disappeared in very early morning (no more than 7 h after the ASP), showing an average duration of almost 4 h (Figure 7). POFs II displayed maximum occurrence also close to the ASP, but they were visible for a longer period of time than in the previous stage. POFs III exhibited a mean duration of 16 h, although they will be present more than 22 h (Figure 7). Resorption stages IV, V and VI had a more continuous distribution than the former stages, appearing in significant abundance throughout the day, although displaying specific periods of maximum and minimum presence (Figure 5A). POFs IV showed an average duration about 30 h, however lasting in the ovary up to more than 37 h. The POFs V and VI had a mean age 32.25 h and 34.54 h, respectively, and both were still recognizable approximately 40 h after ASP (Figure 7). In summary, individuals with ovaries showing follicle resorption until stage III belong to the youngest spawning cohort—they have been caught in the day of the spawning (Day 0). Females with POFs IV to VI belong to a previous spawning cohort (Day 1).

## 4. Discussions

Although observations on reared fishes, including the anchovy, allow for the development of an accurate description and aging of the POF classes (e.g., [7,27]), in other cases, the ability of fish in captivity to produce regular cohorts of POFs may be compromised [35], thus reducing the opportunity of linking POF classification to its age following post ovulation timing. 

The ovaries, collected during the spawning peak of the anchovy, were classified by the identification of the different POF stages, providing clearly recognizable histomorphological features. Among these, the vacuole appearance and the relative effect on the granulosa cells until the breakdown of cell walls, as well as the appearance and proliferation of the nuclear pycnosis were mainly used, as peculiar degradation indices. Similarly, shape (e.g., folder or triangular) and size of the degenerating follicles together with the presence/absence of the lumen were also taken into account. Although some authors considered the position and degradation of the thecal layer not a key discriminating feature [27], in present study they contributed to POF staging. The main cytomorphological modifications above described were already recognized as landmarks for assessing the POF degeneration in European anchovy [27], as well as in several multiple spawning fish species; e.g., northern anchovy [6,11], Pacific sardine (*Sardinops sagax*) [16], Hawaiian anchovy (*Encrasicholina purpurea*) [36], sardine [12,37], chub mackerel (*Scomber japonicus*) [38], Atlantic menhaden (*Brevoortia tyrannus*) [39], and hake (*Merluccius merluccius*) [40]. 

Identification of the POF stages may become easier by the observation of the most developed oocyte stage in the ovary. Generally, early, bigger POFs were detected within gonads showing partially or fully vitellogenic as the most advanced oocyte stage. Later, follicle degeneration proceeds simultaneously to the progressive development of oocytes until ovulation (i.e., hydration); thus, only the last POF stages were occasionally recognized in ovary slides showing nucleus migration as the most advanced oocyte stage. The oocyte maturation reduces the empty space among cells available to host POFs, with a consequent reduction in the follicle sizes. These phenomena induce the acquisition of the triangular-like shape of the late POF stages (Figure 3H), as also described for the sardine [37]. Even so, environmental variability and stress phenomena may condition physiological processes, modifying both the oocyte maturation and the POF resorption [41]. Therefore, the association of the POF stages with the oocyte recruitment might vary seasonally; however, also in literature, the oocyte stages were considered an auxiliary information in POF degeneration evaluation [42]. 

A fast POF resorption at the same time of the oocyte development was already observed and a possible explanation has been linked to continuous oocyte maturation process. Indeed, for an indeterminate spawner, where recruitment of new spawning batches of oocytes occurs continuously, fast resorption is necessary because the aggregation of old POFs would restrict the space available for the development of new oocytes [35,37]. Contrary to later stages (from POF III onwards), present data did not show size shrinkage in the POFs I and II (Figure 4), similar to that observed for anchovy in the Bay of Biscay [27]. In such a population, even an increasing of POF size was recorded from POF I to POF II, but it may be justified by the presence of vacuoles [27], recognised in the literature as a key step in the resorption development process [27,35]. It is likely that the shrinkage due to the resorption process was counterbalanced by the vacuole appearance as well as the modification of the granulosa cells, from columnar to more expanded cubical or spherical aspect (Figure 3A–C). 

The first two stages are bigger than the first ones. Thus, observing the relative occupied areas, younger POFs may appear more abundant than the older, although the former are lower in number. Moreover, younger POFs showed a more rapid degeneration rate that progressively decreased until reaching a minimum value from POFs III onwards (Figure 6). This evidence seems to suggest an “accumulation” phenomenon for older stages, also in accordance with different longevity for each stage. The long presence of stages IV, V and VI in the ovaries may be explained by different temperature encountered by anchovies during the day. Indeed, individuals of the target species displayed a diel vertical migratory behaviour through a wider temperature gradient along thermally stratified water column [13,28]. Furthermore, segregative behaviour of actively spawning females, moving to different bathymetry than the rest of school, was discovered for anchovy stock in the Strait of Sicily [17], as well as for sardines in Atlantic waters [43]. These movements implied a different habitat condition experienced by individuals throughout the day, especially in terms of water temperature. The increasing temperatures determine a decrease in time of POF persistence within the ovaries [39]. In other small pelagics, i.e., sardines, the POF degeneration rate has been observed to grow about 3% per 1 °C in temperature increment [37]. Previous studies already postulated faster POF degeneration during night-time, while the resorption seemed to slow down during daytime when fish remains close to the bottom at lower temperatures [28,44]. 

Histological examination and POF aging results allowed for the identification of two spawning cohorts, i.e., ovaries showing follicle resorption until stage III are considered to belong to the youngest spawning cohort (Day 0), while females with POFs IV to VI concern to a previous spawning cohort (Day 1). Each stage was not considered separate from the following ones because the succession of POFs is a continuous degeneration process with some overlapping among contiguous stages. This may be indicative of the natural variability in the degeneration of POFs among individuals or different conditions encountered by fish. However, present results highlight the importance of validation studies according to different temperature regimes, since POFs in the study area displayed a shorter duration than 3 days until now considered according to studies in Atlantic waters [11], when POF method was applied in the Strait of Sicily. 

Stages I to III showed absences or pronounced minimum occurrences throughout the 24-h cycle, similarly to the anchovy of the Bay of Biscay [13]. Experiments carried out on Atlantic menhaden, tanked at different temperature ranges, demonstrated the initial follicle degeneration between 6 to 12 h after spawning. Later, the transition to successive resorption phases was faster at the warmest temperature [39]. The latter authors postulated that the overlap of the first stages may be due to both variation in degeneration rates as well as differences between the theoretical (estimated) and realized spawning time [39]. Otherwise, the subsequent stages, i.e., POFs IV to VI, appeared recognizable for 24 h in the anchovy in the Strait of Sicily, lasting more than one day and displaying significant occurrences in the night and early morning in agreement with previous results for other Mediterranean populations [28]. 

Differences in total number as well as in the duration of each stage have been observed for this species in Atlantic waters [13]. Furthermore, the same study identified an additional stage, most likely due to the broader persistence of POFs in the ovaries in the cooler Bay of Biscay waters (up to 60 h after spawning at 13 °C–19 °C) compared to the study area, where POFs lasted about 40 h. In the Mediterranean Sea, the little data existing on anchovy POF duration were obtained in the Aegean Sea, during the spawning period with water temperatures between 20 °C and 25 °C [28]. These results were obtained according to the classification based on a different temperature regime (at 13–19 °C; [13]); the authors postulated a POF duration lasting between 32 h and 43 h [28]. 

In other small pelagic species in the eastern Mediterranean waters, full POF resorption was completed in about 58 h at 17 °C–19 °C, as expected for a winter spawning species like the sardine [12]. In Japanese anchovy (*Engraulis japonicus*), postovulatory follicles persisted from 21 h at 25 °C to 34 h at 15 °C [26]. In tropical waters, Hawaiian anchovy POFs last about 24 h after spawning, when surface temperatures are between 20 °C and 29 °C [36], similarly to the maximum duration of POF in skipjack tuna (*Katsuwonus pelamis*) [45], yellowfin tuna (*Thunnus albacares*) [46], and albacore tuna (*Thunnus alalunga*) [47] at the same temperature. POF duration in Atlantic menhaden can vary from 36 h to 60 h, corresponding to a 5 °C range in temperature [39]. In the northern anchovy, Peruvian anchovy, and Pacific sardine, POFs are largely reduced in number and size over 48 h and completely disappeared in 72 h within the temperature 13 °C–19 °C [11]. In Atlantic cod, POFs are easily identifiable 3 months after the spawning season at a temperature lower than 10 °C, while in the common snook (*Centropomus undecimalis*) at 29 °C, the water temperature POFs reached a similar resorption state within 18 h [41]. 

## 5. Conclusions

The degeneration of POFs based on the histomorphological characteristics from field anchovy, covering a 24 h daily cycle, allowed to identify six resorption stages at water temperature around 25 °C, reached in Mediterranean Sea during the anchovy spawning peak. Present results highlighted the presence of two spawning cohort, displaying the persistence of POFs for about one-and-a-half days and no more than two days in anchovy ovaries. This evidence should became a tool for accurate POF cohort identification and then for the spawning frequency estimation, for populations inhabiting regions with higher temperature regimes than the areas (i.e., Atlantic and Pacific) where the most of the previous studies were carried out.

The investigation of POF degeneration is important when DEPM is applied for the fisheries’ independent estimation of spawning biomass [30] because the incorrect age attribution to POFs constitutes major sources of potential bias, determining an overestimation of the biomass, as may have occurred in the case of DEPM application to the anchovy in the central Mediterranean Sea until now. Present results demonstrated the POF degeneration progress at a faster rate than reported by previous investigations, carried out in cooler oceanic waters. According to the POF classification based on the new evaluation of degeneration rate, the spawning fraction estimate should in the future consider a reduced number of days (less than 2 days) of POF persistence than that former adopted (3 days), in agreement with previous studied. These findings should provide methodological advances in estimating the spawning biomass by DEPM and in studying reproductive output fluctuations in the anchovy, particularly for sustainable exploitation purposes. 

## Figures and Tables

**Table 1:** Sampling month, number of anchovy females (No. F), and a number of ovaries (No. Ovaries) histologically examined for postovulatory follicle (POF) evaluation and measuring, per sampling year.

Year	Sampling Period	No. F	No. Ovaries
2008	August	152	11
2009	August	446	29
2012	June–July	474	46
2013	June	298	41
2014	July	222	44
2015	July	516	74

**Table 2:** Number of catches (No. Catches) and number of postovulatory follicles (No. POF) per two hour-long intervals.

Time	No. Catches	No. POF
00:00–01:59	4	37
02:00–03:59	4	34
04:00–05:59	33	369
06:00–07:59	8	47
08:00–09:59	50	471
10:00–11:59	25	158
12:00–13:59	32	208
14:00–15:59	38	262
16:00–17:59	27	197
18:00–19:59	9	47
20:00–21:59	8	54
22:00–23:59	8	54

**Table 3:** Evaluation of difference in mean size of postovulatory follicles (POF) stage. Significance of differences has been evaluated for each stage (POF I to POF VI) by Kruskal–Wallis test: H (5, n = 1940) = 1056.59 *p* < 0.001.

POF Stage	POF I	POF II	POF III	POF IV	POF V	POF VI
**POF I**		1.00	1.00	0.000459	0.00	0.00
**POF II**			1.00	0.007490	0.00	0.00
**POF III**				0.000001	0.00	0.00
**POF IV**					0.00	0.00
**POF V**						0.00
**POF VI**						

## Data Availability

The data presented in this study are available on request from the corresponding author.

## Supplementary Materials

The following are available online at https://www.mdpi.com/2076-2615/11/2/529/s1, Figure S1: Details on evolution of the postovulatory follicle (POF) resorption process as well as 4 the most important histo-morphological features for each stage.
