# Supplementary material for: New Evaluation of Postovulatory Follicle Degeneration at High-Temperature Regimes Refines Criteria for the Identification of Spawning Cohorts in the European Anchovy (Engraulis encrasicolus)
Source: Animals (Basel). 2021 Feb 18;11(2):529. doi: 10.3390/ani11020529 (PMC7922735; doi:10.3390/ani11020529)
Supplement: Supplementary file 1 [file animals-11-00529-s001.pdf]

animals

MDPI

Supplementary materials to Ferreri et al. "New evaluation of postovulatory follicle degeneration at high temperature regimes refines criteria for the identification of spawning cohorts in the European anchovy (*Engraulis encrasicolus*)".

Details on evolution of the postovulatory follicle (POF) resorption process as well as the most important histo-morphological features for each stage are showed in the pictures below (Figure S1A-H).

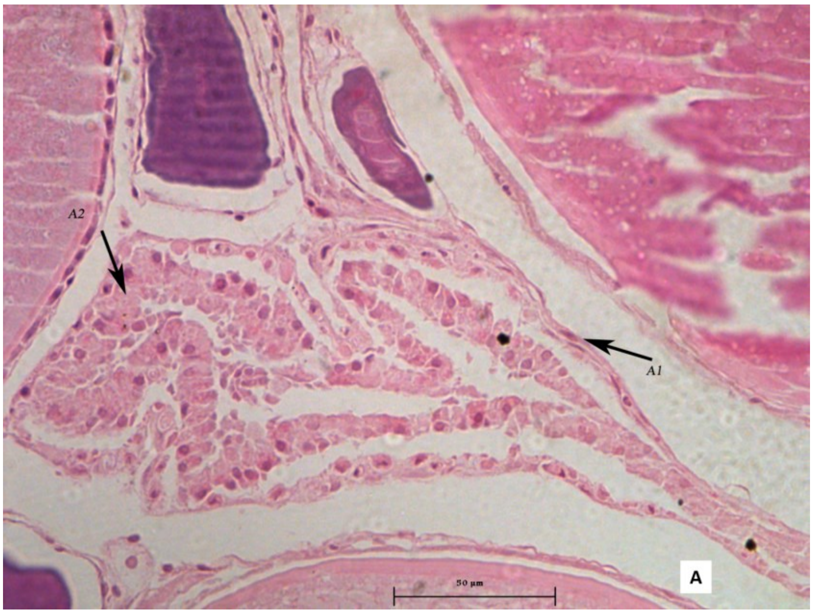

Figure S1A. *POF STAGE I.* The whole POF structure is strictly folder, showing big size, irregular shape and irregular shaped lumen. Black arrows: A1, cell membranes well visible; A2, granulosa cells appeared compact with a good level of alignment. Scale bar = 50 µm.

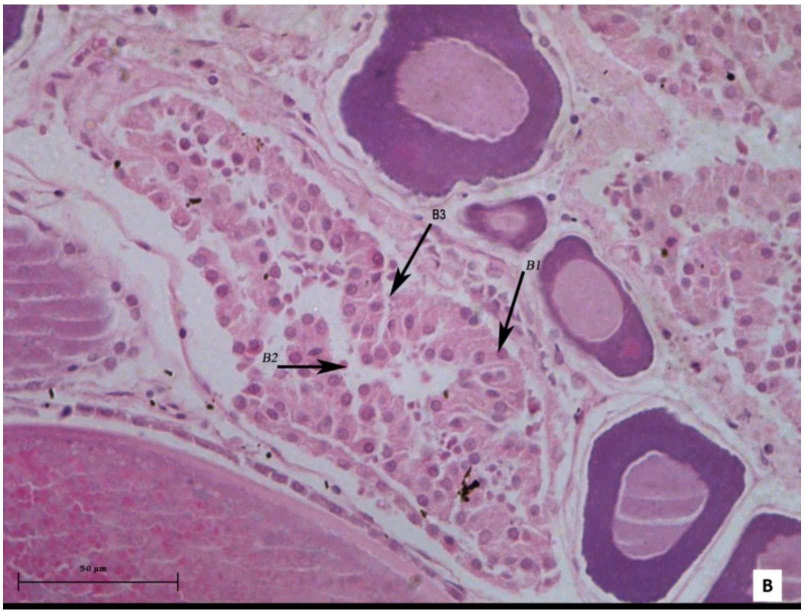

Figure S1B. *POF STAGE I (Bis).* The POF showed very early evidences of degeneration structure. Black arrows: B1, The membrane cells appear extended due to the extensively elongation experienced during the hydration phenomenon; B2, irregular-shaped lumen; B3, the granulosa determine the strictly folder, irregular shape. Scale bar = 50 µm.

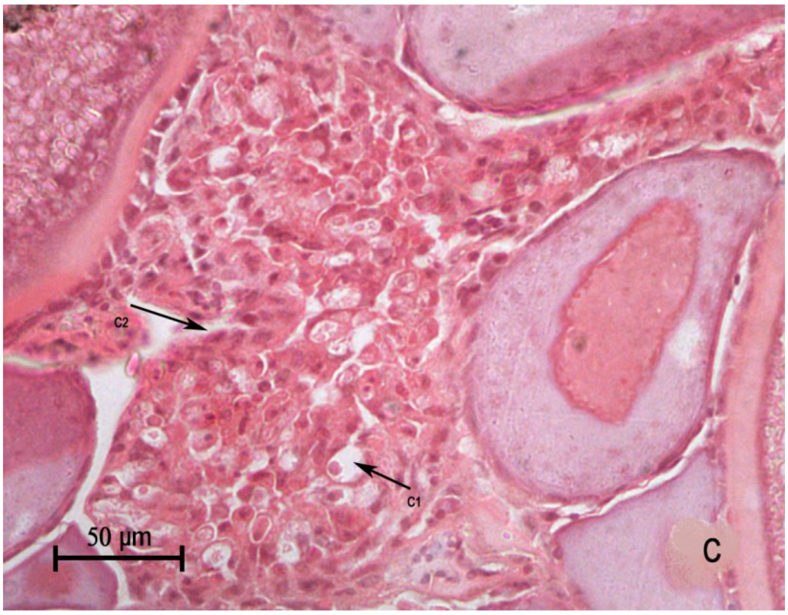

Figure S1C. *POF STAGE II***.** The POF showed big size. Black arrows: C1, vacuoles, which appearance represent the structure discriminating for stage II; C2, early evidences of cellular deterioration are visible. Scale bar = 50 µm.

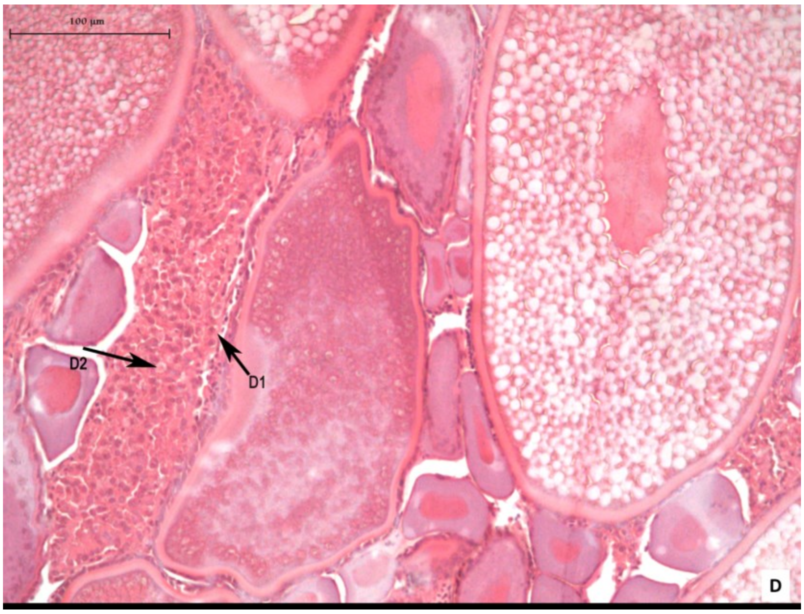

Figure S1D. *POF STAGE III.* The POF in this stage displayed a reduction of extension and a lumen shrinkage. Black arrows: D1, the granulosa cells showed a spherical shape; D2, numeros vacuoles are visible. Scale bar = 100 µm.

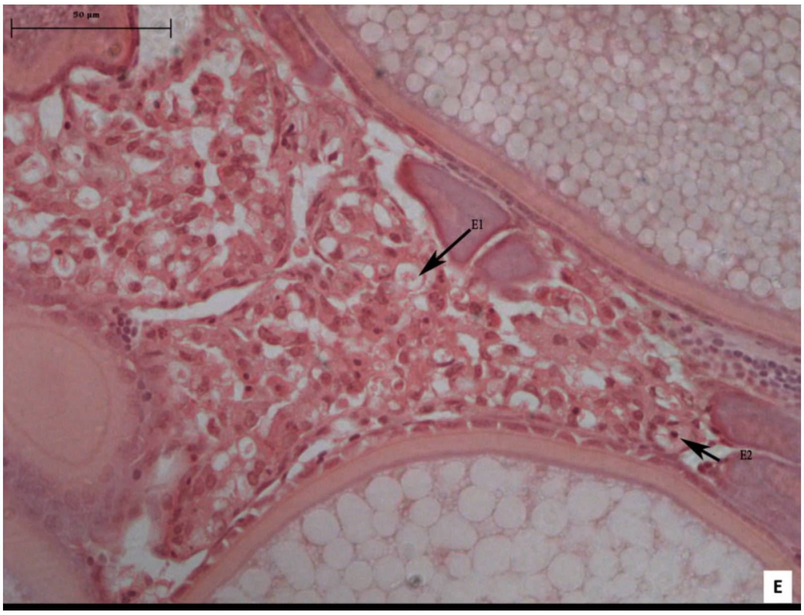

Figure S1E. *POF STAGE IV.* In this figure, two POFs are visible. The POF IV showed a compacted, flat aspect, as well as reduced extension. Black arrows: E1, The vacuoles, still abundant and well visible, induce the complete breakdown of the cellular walls; E2, appearance of picnotic nuclei. Scale bar = 50 μm.

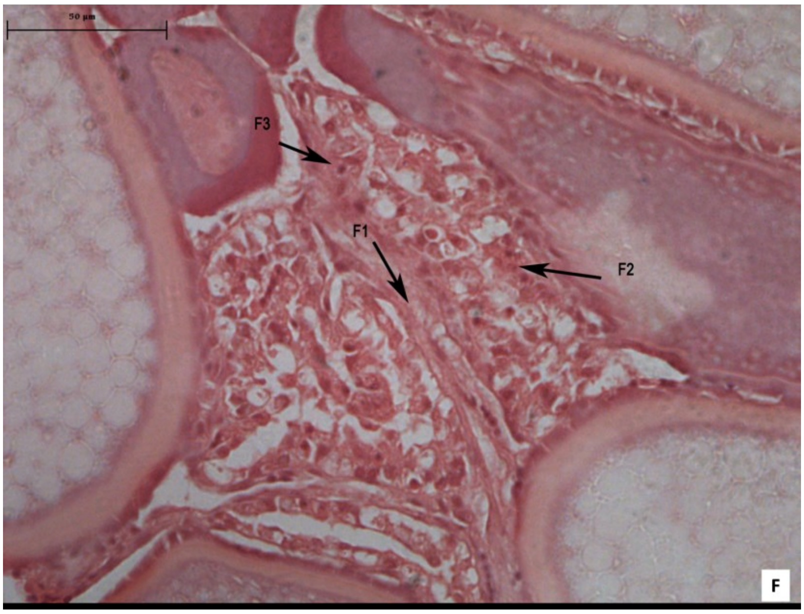

Figure S1F*. POF STAGE V*. The POF looked very compact, shrunk. Black arrows: F1, the lumen is not visible anymore; F2, the nuclei are identifiable, although picnotic; F3, The theca may be occasionally present, but not easily distinguishable. Scale bar = 50 μm.

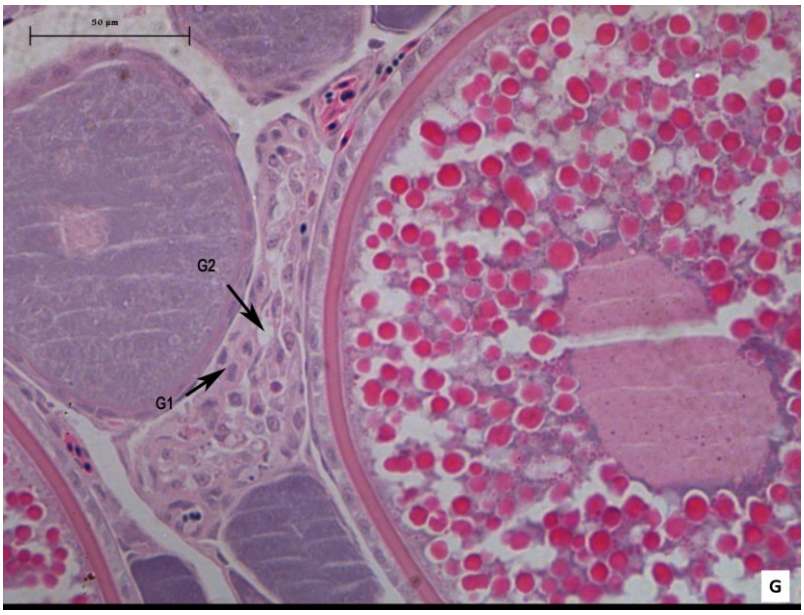

Figure S1G. *POF STAGE VI.* This last resorption POF stage showed small extension and, generally, an almost triangular, elongated shape. The follicles resulted amorphous, disorganized, without clear, distinguishable cellular structures. Black arrows: G1, all the nuclei are picnotic; G2, very few vacuoles are still visible. Scale bar = 50 µm.

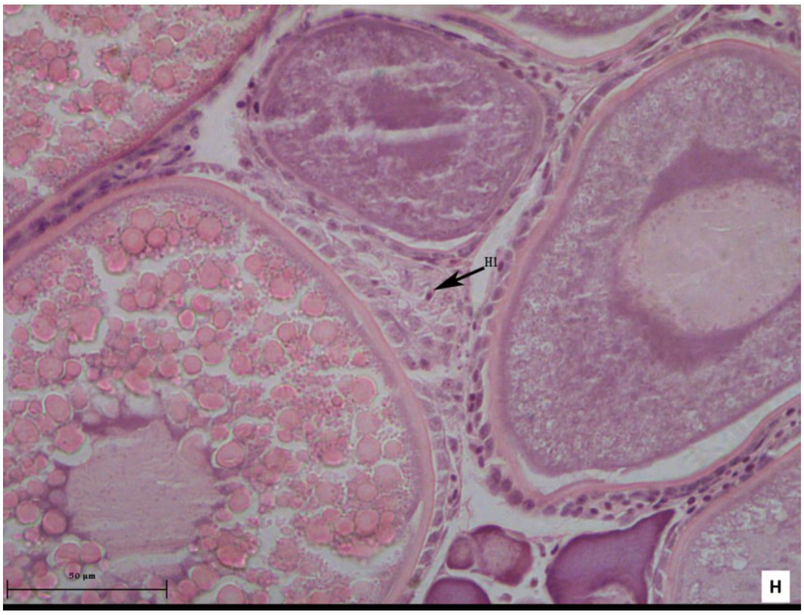

Figure S1H. *POF STAGE VI (Bis).* This last resorption POF stage showed small extension and, generally, an almost triangular, elongated shape. The follicles resulted amorphous, disorganized, without clear, distinguishable cellular structures. No cellular differentiation is visible neither the theca layer. Black arrows: H1, some picnotic nucleus may be still observable. Scale bar = 50 µm.
